# Selected physical activities and the risk of endometrial cancer.

**DOI:** 10.1038/bjc.1993.155

**Published:** 1993-04

**Authors:** F. Levi, C. La Vecchia, E. Negri, S. Franceschi

**Affiliations:** Registre Vaudois des Tumeurs, CHUV, Lausanne, Switzerland.

## Abstract

The relationship between various indicators of physical activity and endometrial cancer risk was analysed using data of a case-control study conducted in 1988-1991 in Switzerland and Italy on 274 histologically confirmed cases and 572 controls admitted to hospital for acute, non neoplastic, non hormone-related diseases. Using a self-rated assessment of total physical activity, there was a systematic tendency for the cases to report more frequently 'low' or 'very low' physical activity. The relative risks were similar for 'very high' or 'moderately high' physical activity, but increased in the two lowest levels, with point estimates, in various decades of age, between 1.3 and 2.3 for 'moderately low' and over 2.5 for 'very low' physical activity. Although the association was apparently stronger at older ages, all the trends in risk were significant. Allowance for major identified potential distorting factors, including body mass index and a measure of total energy intake, could explain only in part the association, and the inverse trends in risk remained statistically significant. When selected types of physical activity were analysed, no association was observed with climbing stairs or walking, but the risk estimates for the lowest level of activity was over 4 for housework, and between 1.5 and 1.9 for sport and leisure and occupational activity. Thus, the present findings suggest that a moderate or high physical activity is an indicator of reduced endometrial cancer risk, although this observation still requires epidemiologic confirmation and clearer definition from a pathogenic point of view.


					
Br. J. Cancer (1993), 67, 846 851                                                                       ?  Macmillan Press Ltd., 1993

Selected physical activities and the risk of endometrial cancer

F. Levi',2, C. La Vecchia2'3, E. Negri3 & S. Franceschi4

'Registre Vaudois des Tumeurs, CHUV-Falaises 1, 1011 Lausanne; 2Institute of Social and Preventive Medicine, University of
Lausanne, 1005 Lausanne Switzerland; 3Istituto di Ricerche Farmacologiche 'Mario Negri', 20157 Milano; 4Servizio di
Epidemiologia, Centro di Riferimento Oncologico, 33081 Aviano (PN), Italy.

Summary The relationship between various indicators of physical activity and endometrial cancer risk was
analysed using data of a case-control study conducted in 1988-1991 in Switzerland and Italy on 274
histologically confirmed cases and 572 controls admitted to hospital for acute, non neoplastic, non hormone-
related diseases. Using a self-rated assessment of total physical activity, there was a systematic tendency for the
cases to report more frequently 'low' or 'very low' physical activity. The relative risks were similar for 'very
high' or 'moderately high' physical activity, but increased in the two lowest levels, with point estimates, in
various decades of age, between 1.3 and 2.3 for 'moderately low' and over 2.5 for 'very low' physical activity.
Although the association was apparently stronger at older ages, all the trends in risk were significant.
Allowance for major identified potential distorting factors, including body mass index and a measure of total
energy intake, could explain only in part the association, and the inverse trends in risk remained statistically
significant. When selected types of physical activity were analysed, no association was observed with climbing
stairs or walking, but the risk estimates for the lowest level of activity was over 4 for housework, and between
1.5 and 1.9 for sport and leisure and occupational activity. Thus, the present findings suggest that a moderate
or high physical activity is an indicator of reduced endometrial cancer risk, although this observation still
requires epidemiologic confirmation and clearer definition from a pathogenic point of view.

Endometrial cancer is strongly related to overweight and,
possibly, to selected aspects of dietary intake (La Vecchia,
1989; Parazzini et al., 1991). Physical activity is the other
main component of energy balance, and appears to exert a
favourable effect on a number of chronic conditions, includ-
ing  coronary   heart  disease,  diabetes  mellitus,  and
osteoporosis (Garfinkel & Stellman, 1988; Helmrich et al.,
1991). For instance, several studies of coronary heart disease
have found protections of the order of 30 to 60% for sub-
jects reporting at least moderate physical activity, and the
effect of exercise was apparently independent from that of
other recognised risk factors for heart disease (Morris et al.,
1973; Paffenbarger & Hale, 1975; Paffenbarger et al., 1978;
Garfinkel & Stellman, 1988). An association of inactivity or
low physical activity with large bowel cancer has also been
reported, and was apparently independent from body mass
index, serum cholesterol, alcohol and other identified or
potential confounding factors (Ballard-Barbash et al., 1990;
Whittemore et al., 1990).

It has been suggested that physical activity may modify
serum cholesterol levels (Caspersen et al., 1991), amount and
distribution of body fat (Seidell et al., 1991), the ovarian
production of female hormones (Frisch et al., 1987) and
serum oestrogen levels in postmenopausal women (Cauley et
al., 1989). These may influence some of the biologic links
between anthropometric and hormonal factors and endo-
metrial cancer risk (La Vecchia, 1989).

With these background considerations, albeit aware of the
major difficulties both in the reliable assessment of physical
exercise (Washburn & Montoye, 1986) and in sensible
allowance for covariates, we considered the relationship
between selected measures of physical activity and endo-
metrial cancer risk using data from a case-control study
conducted in Switzerland and Italy.

Subjects and methods

Since January 1988, we have been conducting a cooperative
case-control study of endometrial cancer in the Swiss Canton

of Vaud, and in metropolitan Milan and in Pordenone pro-
vince, northern Italy. The general design of this investigation
has already been described (Levi et al., 1992).

Briefly, in Vaud case recruitment was centered in the main
University Hospital of the Canton, and cases were matched
with incident cases reported to the local Cancer Registry
(Levi, 1987). Overall, over 80% of identified cases were inter-
viewed. In Milan and Pordenone case recruitment was
hospital-based, since the areas are not covered by cancer
registration schemes. Overall, 274 histologically confirmed
incident endometrial cancers were interviewed before June,
1991. The age range was 31-75 (median age 61 years).

The controls were women aged 75 or less, admitted to the
same networks of hospitals where cases had been identified,
whose primary diagnosis was unrelated to any of the known
or potential risk factors for endometrial cancer or to any
long-term modification of diet. Specific exclusion was made
of women admitted for gynaecologic, hormonal, metabolic or
neoplastic diseases or who had undergone hysterectomy. A
total of 572 women, aged 30 to 75 (median age 59), were
interviewed. Of them, 32% were admitted for traumas, 16%
had non-traumatic orthopaedic diseases, 26% surgical con-
ditions and 26% other miscellaneous disorders, including
acute medical, eye, nose and throat and dental diseases, etc.
Cases and controls were not singularly matched, but, to
avoid major imbalances in the age distribution, controls were
frequency matched with cases within each centre.

All interviews were conducted in hospital by trained inter-
viewers (nurses or medical students). Approximate length of
the interview was between 40 min and 1 h. Less than 10% of
subjects (7% of cases, 8% of controls) approached for inter-
view refused. The main reason for non-response was absence
from the ward during the interviewers' visit. The interviewers
received the same structured training, to standardise data
collection in various centres. Data checking and quality con-
trol was centralised.

Information was collected using the same structured
questionnaire on socio-demographic factors, personal charac-
teristics and habits (such as smoking, coffee and alcohol
drinking), self-reported current and past anthropometric
measures (Levi et al., 1992), 40 selected indicator foods, a
problem-oriented medical history, menstrual and reproduc-
tive factors and use of female hormone preparations. A few
indicators of physical activity were also elicited including, for
various ages, an overall subjective (self-rated) score for total

Correspondence: F. Levi.

Received 16 April 1992; and in revised form 16 November 1992.

'?" Macmillan Press Ltd., 1993

Br. J. Cancer (1993), 67, 846-851

PHYSICAL ACTIVITY AND ENDOMETRIAL CANCER 847

activity arbitrarily subdivided into four levels (high/moderately
high/moderately low/very low) and based only on patients'
evaluation and report, plus five selected types of activities
(housework, climbing stairs, walking, sport and leisure,
occupational activity), again assessed in four levels on the
basis of quantity (high/moderately high/moderately low/low)
and frequency (daily/weekly/occasionally/never).

Data analysis and control of confounding

Relative risks (RR), and the corresponding 95% confidence
intervals (CI) (Breslow & Day, 1980) of endometrial cancer
according to various indicators of physical activity were first
computed from data stratified for study centre and 5-year age
group using the Mantel-Haenszel procedure (Mantel &
Haenszel, 1959). Further, multiple logistic regression was
used, with maximum likelihood fitting (Baker & Nelder,
1976), including terms for study centre, age, education,
parity, menopausal status, use of oral contraceptives and
oestrogen replacement treatment, body mass index and
estimated total calorie intake. This was computed by multi-
plying the average caloric content of one serving of each of
the 40 foods and of beverages (including alcoholic beverages)
by its reported frequency of intake, using nutritional values
issued by English (Paul & Southgate, 1978) or Italian (Fidanza
& Versiglioni, 1988) tables. Estimated average total calorie
intake for the overall dataset was 1,980 Kcalories per day.

Results

Table I gives the distribution of endometrial cancer cases
and the comparison group according to study centre, age
group, menopausal status, and body mass index. Cases were
somewhat older than controls, more frequently in post

menopause, of similar level of education, but had
significantly greater body mass: compared with normal-
weight women, the relative risk was 1.4 (95% CI 1.0 to 2.0)
for overweight ones, and 2.4 (95% CI 1.3 to 5.1) for the
severely obese).

Table II considers an overall subjective score of total
physical activity at different ages. In each subsequent age
group there was a systematic tendency for the cases to report
more frequently 'low' or 'very low' physical activity. The
corresponding relative risk estimates are given in Table III.
The RRs were similar for 'very high' and 'moderately high'
physical activity, but increased in the two lowest levels, with
point estimates between 1.3 and 2.3 for 'moderately low'
overall physical activity, and over 2.5 for 'very low' physical
activity. Although the association was apparently stronger at
older ages, all the trends in risk were significant. Allowance
for major identified potential distorting factors, including
body mass index and a measure of total energy intake, could
explain only in part the association with total physical
activity, and all the inverse trends in risk remained statis-
tically significant, indicating that physical activity had an
independent effect from main recognised risk factors for
endometrial cancer.

Selected types of physical activity (housework, climbing
stairs, walking, sport and leisure, and occupational activity)
during the decade of age before diagnosis or interview are
considered in Table IV in terms of frequency distribution of
cases and controls and in Table V in terms of relative risk
estimates. No association was observed with climbing stairs
or walking, but housework, sport and leisure, and occupa-
tional activities were inversely and significantly related with
endometrial cancer. The risk estimates for the lowest level of
activity were over 4 for housework, and between 1.5 and 1.9
for sport and leisure and occupational activity. As for total
activity, all trends in risk were not appreciably modified after

Table I Distribution of 274 cases of endometrial cancer and 572 controls
according to study centre age, menopausal status, and current body mass

index

Endometrial cancer          Controls

Number        %         Number        %
Study centre

Vaud                      138         50.4       406         71.0
Milan                     112        40.9         122        21.3
Pordenone                  24         8.8         44          7.7
Age group (years)

<45                        16         5.8         65         11.4
45-54                      51         18.6        128        22.4
55-64                     112        40.9        187         32.7
65-75                      95        34.7         192        33.6
Menopausal status

Pre- in menopause          56        20.4         156        27.3
Post-menopause            218         79.6       416         72.7
Current body mass index

(kg m-2)

<20                        17         6.2         60         10.5
20-24                     112        40.9         253        44.2
25-29                      93         33.9        190        33.2
> 30                       52        19.0         69         12.1

Table II Distribution of 274 cases of endometrial cancer and 572 controls according to reported measures of total physical activity at different

ages

Age (years)

25                 35                45                 55                 65

Cases   Controls   Cases   Controls  Cases    Controls  Cases   Controls   Cases   Controls
Measure of physical activity

1 (highest)                        111      247       105      246       88       206       53       121      17        45
2                                   99      258       101      261       112      261      121      247       66       147
3                                   50       57        53       53       50        61       46        70      47        77
4 (lowest)                          14        10       14       11        17       12       23         9      23         8

848    F. LEVI et al.

00

C14   C?i       00

OR

CY-,v                                                                                 ON en     WI

C4 tn                                                                                 00 en

en

Iq    Wi 06

Cd                                                                        eq lqt 00 00
0?

C4 C4

1 6
t-    r. oo <?

en                                                                             z

h.     ,q, 00 as

en C14 4T No

C'4

rA

en

en

ed

Cd                                                                                                                            C14 as 4n ?o

0                                 tn    ON

ON 1,0   ?o

C4    ?6                                                                                       00 CYN t-

I     I      I   tl-

0

C? In      rA

Ca

NO

No W) en

C?    06       0  ed

eli   00

A:6 co

C;N       en    W;                                                                        bo

I

Ci

r4 C14 ?Q Rt

C,4 mt a.,
Cd

r.
00

en                                                                                                en    tl-

C-i

'Itt ?o ON %n

ON kn so
00

WI

lz

W)                                C.,    C14

ON                                C14               *-A

00    oo                                                                                          Zt,
C;    -4 el C4             0

Cd                                                 (u

>

(U

>

C14 en IRt
co                                                                                                                        t3

Z

Cd
N.-

04
0

PHYSICAL ACTIVITY AND ENDOMETRIAL CANCER  849

C- l  C l  m lr-

10 I    -
0 0

0q   0-

- 0

C c  l  C l  l C

o  o  I  I

00 0

CC
- 4 4o%

6     6

1-    I.-    .1-

Cl     Cl      r en

-          Clo as6 -nc
46 c            00

0         - 0   0   I

-   Cl  4- t
X  oW I I  I  o  I  m

0  O - 90   (  0 m

6-o

5   1-  CD

Cl - -0%

X Cl a - l C l 9

0 - 0%

Cl - - s

a- Cou - t

a-  e   -
-   -  C

X   .   l  .  Cl

-   -  Cl

U~~~~~~~~

0         ?

0
X  , Io

- o - N t '0

-      a

.0

' 0

: 0

allowance for major identified potential confounding factors.

Similar results were obtained when the same indicators of
physical activity were considered across subsequent age
groups. Likewise, similar analyses conducted in the two study
areas separately (Switzerland/Northern Italy) yielded com-
parable results (data not shown).

Table VI considers the interaction between estimated total
physical activity, body mass index and total calorie intake.
There was a suggestion for the association to be stronger in
heavier women and in those who reported higher calorie
intake, although the point estimates for the lowest level of
physical activity tended to be above unity in most strata
considered. The interaction term was significant only for
body mass index (x2= 12.33, P<0.01).

Discussion

The present study suggests that physical activity is an
indicator of reduced endometrial cancer risk. The association
was evident and consistent with an overall self-rated assess-
ment of total physical activity across various periods of life,
and with a few selected types of physical activities
(housework, sport and leisure, occupational activity), which
probably represent the indicators of physical activity with the
best discriminating ability. The observed association was not
explained by major established risk factors for endometrial
cancer and other main correlates of energy balance, including
calorie intake and body mass index.

If the strength and the consistency of the results allow to
exclude confidently chance or obvious bias as an explanation
of the associations observed, it is nonetheless difficult to
formulate and discuss any inference on a causal role of
exercise on endometrial carcinogenesis. Epidemiologically,
there are major difficulties in studying exercise (Washburn &
Montoye, 1986). Occupational exercise, in fact, is strongly
correlated to social class and hence to a wide range of other
environmental and lifestyle exposures. Leisure time exercise is
also confounded, though to a lesser extent, by social class
indicators, and can be a consequence, as well as a cause, of
general health conditions.

To further complicate the issues, physical activity is
difficult to quantify, and the related methods of validation
and analysis are still open to discussion (Washburn & Mon-
toye, 1986). Although the assessment of physical activity
through questionnaires is still open to debate, it has been
suggested that even simple questions provide useful inform-
ation on the issue (Schechtman et al., 1991). We used
therefore a simplified overall subjective score for physical
activity, plus a few specific questions on a number of selected
types of activities which are commonest in the study popula-
tion. Although each one of these measures may be open to
criticism, the consistency of the findings with reference to
several different types of physical activity lends support to
the validity of the results.

Further difficulties are posed by the lack of a simple
biologic interpretation of the observed association. It has
been suggested that physical activity decreases body fat
(independently from potential modifications of body mass
index (Frisch et al., 1987)) and influence body fat distribution
(Seidell et al., 1991) and serum lipoprotein levels (Caspersen
et al., 1991). Another plausible biologic link would be
through serum oestrogen levels, since more active women had
been found to have lower levels of oestrone (Cauley et al.,
1989) or increased metabolism of oestrogens to less potent
forms (Frisch et al., 1985), and this observation is consistent
with the especially stronger association observed in this study

'e

0

o

-

Cd

a

04

a

a)

in obese women and in those reporting higher calorie intake.

A few studies have suggested an inverse relationship
between physical activity and colorectal (Whittemore et al.,
1990), breast and female genital tract cancer risk (Frisch et
al., 1987). However, the favourable breast cancer risk pattern
among ballet dancers (as indicated by delayed menarche and
amenorrhea (Frisch et al., 1980, 1981)) and other groups of
women with high physical activity in adolescence has been

s- *-i
Ci  .3

;E q
(Z

Cd

a

-

0

c)

*-0

a)
0
CO
a
0

CZ

. -

B

c)

-

ai
a

a)

'0

a
0

850    F. LEVI et al.

Table VI Relative risk estimates (and 95% confidence intervals)' of endometrial cancer according to
reported measures of total physical activity in selected strata of body mass index and total calorie

intake

Total physical activityb

JC           2            3           4            X2

Variable                          (highest)                             (lowest)     (trend)
Body mass index kgm2

< 25                                1          0.7          0.6          1.8         0.08

(0.4-1.2)    (0.3-1.1)   (0.6-5.2)

25                                1           1.0          1.8         4.7         14.38d

(0.6-1.8)    (0.9-3.4)   (1.9-11.3)
Total calorie intake, tertile'

1 (lowest)                         1           0.9          0.6          1.0         0.26

(0.4-2.1)    (0.2-1.7)   (0.2-3.7)

2                                   1          0.7          1.0          2.3         0.85

(0.3-1.4)    (0.5-2.2)   (0.5-9.8)

3 (highest)                         1          0.7          0.9          3.7         3.71

(0.4-1.5)    (0.4-2.0)   (1.2-11.3)

'Mantel-Haenszel estimates adjusted for study centre and age. bIn the decade of age before
diagnosis/incidence.cReference category.dP<0.01.eCut off points, 1,782, 2,188 Kcalories/day.

generally interpreted in terms of the effect of thinness (and
related hormonal factors), rather than of a direct influence of
physical activity (Frisch et al., 1987; La Vecchia, 1989).

To further complicate any inference, some other epidemio-
logic observations are inconsistent with the hypothesis that
physical exercise reduces overall cancer incidence. The
American   Cancer Society  One   Million  Cohort Study
(Garfinkel & Stellman, 1988), for instance, found reduced
standardised mortality ratios (SMR) for all causes and
ischaemic heart disease in subjects reporting heavy exercise,
but elevated rates in women (SMR = 120) for all cancers.
Likewise, in a cohort study of over 50,000 alumni, physical
activity (  5 h per week) was associated with reduced rectal
cancer risk (RR = 0.6, 95% CI 0.2-1.0), but no consistent
protection for other cancer sites (Albanes et al., 1989). Again,
confounding cannot be excluded, but these data do not
support the view that physical exercise per se reduces cancer
risk. The NHANES I cohort study (Albanes et al., 1989)
showed that the risks of all cancers, colorectal and lung
cancers were elevated among inactive males, when non-
recreational activity was considered, but the pattern was less
consistent for females, with an increased risk of cervical
cancer among inactive women and a non-significant associa-
tion between inactivity and postmenopausal breast cancer.

Other limitations and strengths of this study are common
to most hospital-based case-control investigations (Mantel &
Haenszel, 1959; Breslow & Day, 1980). Although hospital
controls can in principle be criticized for the investigation of
lifestyle habits, it is unlikely that patients admitted to hos-
pital for a wide spectrum of acute diseases reported
systematically more physical activity than the general popula-
tion. Separate comparison with various diagnostic categories
of the controls, moreover, did not find any appreciable
difference in any of the risk estimates. Selection, information,
or confounding bias are unlikely to play a major role, since

participation rate was almost complete, the catchment areas
of cases and controls were comparable, and there is no reason
for suggesting differential recall of physical exercise by cases
and controls. In addition, allowance for a number of major
covariates in multivariate analyses did not appreciably
modify any of the risk estimates.

In conclusion, therefore, the present study suggests that a
moderate or high physical activity is an indicator of protec-
tion against endometrial cancer risk, although the observa-
tion still requires epidemiologic confirmation and also
pathogenetic interpretation to be integrated within our
knowledge of endometrial carcinogenesis.

The study was supported by the Swiss National Science Foundation
Grant 32.9495.88 and the Italian National Research Council (CNR),
Applied Projects 'Clinical Applications of Oncological Research'
(Contract No. 92.02384. 8F 39) and 'Prevention and Control of
Disease Factors' (Contract No. 91.00285.8141). The contributions of
the Swiss and Italian Leagues against Cancer, the Italian Association
for Cancer Research and of the 'Europe Against Cancer' Programme
of the Commissions of the European Communities are gratefully
acknowledged. The authors thank Ms C. Gulie and Dr V. Monnier,
for data collection, hospital physicians Dr H. Bossart, Dr P. Bur-
ckhardt, Dr G. Chapuis, Dr P. De Grandi, Dr N. De Tribolet, Dr
J.-F. Delaloye, Dr E. Frenk, Dr S. Krupp, Dr S. Leyvraz, Dr J.-J.
Livio, Dr P. Magnenat, Dr R. Mirimanoff, Dr R. Mosimann, Dr P.
Nicod, Dr M. Savary (Centre Hospitalier Universitaire Vaudois,
Lausanne), Dr Cl. Gailloud and Dr L. Zografos (H6pital Ophtalmi-
que, Lausanne); practitioners Dr H. Achtari, Dr J.-F. Bauer, Dr R.
Born, Dr Y. Csank, Dr A. Curchod, Dr B. Diserens, Dr P.-J.
Ditesheim, Dr P.-J. Dubuis, Dr R. Flury, Dr H.-D. Getaz, Dr A.
Heim, Dr G. Isler-de Gunten, Dr Ph. Koch, Dr S. Meyer, Dr J.-F.
Monod, Dr L. Morard, Dr A. Noyer, Dr P. Pugin, Dr J.-F. Rossat,
Dr A. Scheggia, Dr U. Stoll, Dr D. Vajda, Dr H.-C. Viscolo, Dr R.
Wermelinger, Dr E. Wilson; the Vaud Cancer Registry's staff (Mrs
F. Golay, Mrs N. Menoud, and Dr V.-C. Te) and Mrs H.-C. Janin
for editorial assistance.

References

ALBANES, D., BLAIR, A. & TAYLOR, P.R. (1989). Physical activity

and risk of cancer in the NHANES I population. Am. J. Public
Health, 79, 744-750.

BAKER, R.J. & NELDER, J.A. (1976). The GLIM System Release 3.

Numerical Algorithms Group: Oxford.

BALLARD-BARBASH, R., SCHATZKIN, A., ALBANES, D., SCHIFF-

MAN, M.H., KREGER, B.E., KANNEL, W.B., ANDERSON, K.M. &
HELSEL, W.E. (1990). Physical activity and risk of large bowel
cancer in the Framingham Study. Cancer Res., 50, 3610-3613.
BRESLOW, N.E. & DAY, N.E. (1980). Statistical Methods in Cancer

Research. Vol. 1. The analysis of case control studies. Interna-
tional Agency for Research on Cancer, IARC Scientific Publica-
tions No. 32: Lyon.

CASPERSEN, C.J., BLOEMBERG, B.P.M., SARIS, W.H.M., MERRITT,

R.K. & KROMHOUT, D. (1991). The prevalence of selected
physical activities and their relation with coronary heart disease
risk factors in elderly men: the Zutphen study, 1985. Am. J.
Epidemiol., 133, 1078-1092.

CAULEY, J.A., GUTAI, J.P., KULLER, L.H., LEDONNE, D. & POWELL,

J.G. (1989). The epidemiology of serum sex hormones in post-
menopausal women. Am. J. Epidemiol., 129, 1120-1131.

FIDANZA, F. & VERSIGLIONI, N. (1988). Tabelle di composizione

degli alimenti. In Nutrizione umana, Fidanza, F. & Liguori, G.
(eds) pp. 677-730. Idelson: Naples.

PHYSICAL ACTIVITY AND ENDOMETRIAL CANCER  851

FRISCH, R.E., WYSHAK, G. & VINCENT, L. (1980). Delayed menar-

che and amenorrhea in ballet dancers. N. Engi. J. Med., 303,
17-19.

FRISCH, R.E., GOTZ-WELBERGEN, A.V., McARTHUR, J.W. & 6

others (1981). Delayed menarche and amenorrhea of college ath-
letes in relation to age of onset of training. JAMA, 246,
1559-1563.

FRISCH, R.E., WYSHAK, G., ALBRIGHT, N.L. & 7 others. (1985).

Lower prevalence of breast cancer and cancers of the reproduc-
tive system among former college athletes compared to non-
athletes. Br. J. Cancer, 52, 885-891.

FRISCH, R.E., WYSHAK, G., ALBRIGHT, N.L., ALBRIGHT, T.E.,

SCHIFF, I., WITSCHI, J. & MARGUGLIO, M. (1987). Lower
lifetime occurrence of breast cancer and cancers of the reproduc-
tive system among former college athletes. Am. J. Clin. Nutr., 45,
328-335.

GARFINKEL, L. & STELLMAN, S.D. (1988). Mortality by relative

weight and exercise. Cancer, 62, 1844-1850.

HELMRICH, S.P., RAGLAND, D.R., LEUNG, R.W., PAFFENBARGER,

A.B. & PAFFENBARGER, R.S. Jr. (1991). Physical activity and
reduced occurrence of non-insulin-dependent diabetes mellitus. N.
Engl. J. Med., 325, 147-152.

LA VECCHIA, C. (1989). Nutritional factors and cancers of the breast,

endometrium and ovary. Eur. J. Cancer Clin. Oncol., 25,
1945- 1951.

LEVI, F. (1987). Statistics from the registry of the canton of Vaud,

Switzerland, 1978-1982. In Cancer Incidence in Five Continents,
Muir, C.S., Waterhouse, J., Mack, T., Powell, J. & Whelan, S.
(eds) IARC Scientific Publications No. 88, Vol. V, pp. 634-639:
Lyon.

LEVI, F., LA VECCHIA, C., NEGRI, E. & FRANCESCHI, S. (1992).

Body mass at different ages and subsequent endometrial cancer
risk. Int. J. Cancer, 50, 567-571.

MANTEL, N. & HAENSZEL, W. (1959). Statistical aspects of the

analysis of data from retrospective studies of disease. JNCI, 22,
719-748.

MORRIS, J.N., CHAVE, S.P.W., ADAM, C., SIREY, C. & EPSTEIN, L.

(1973). Vigorous exercise in leisure-time and the incidence of
coronary heart-disease. Lancet, 1, 333-339.

PAFFENBARGER, R.S. Jr. & HALE, W.E. (1975). Work activity and

coronary heart mortality. N. Engl. J. Med., 292, 545-550.

PAFFENBARGER, R.S. Jr., WING, A.L. & HYDE, R.T. (1978). Physical

activity as an index of heart attack risk in college Alumni. Am. J.
Epidemiol., 108, 161-175.

PARAZZINI, F., LA VECCHIA, C., BOCCIOLONE, L. & FRANCESCHI,

S. (1991). The epidemiology of endometrial cancer. Gynecol.
Oncol., 41, 411-416.

PAUL, A.A. & SOUTHGATE, D.A.T. (1978). McCance and Widdow-

son's the Composition of Foods. 4th ed. Medical Research Coun-
cil: London.

SCHECHTMAN, K.B., BARZILAI, B., ROST, K. & FISCHER, E.B. Jr.

(1991). Measuring physical activity with a single question. Am. J.
Public Health, 81, 771-773.

SEIDELL, J.C., CIGOLINI, M., DESLYPERE, J.P., CHARZEWSKA, J.,

ELLSINGER, B.M. & CRUZ, A. (1991). Body fat distribution in
relation to physical activity and smoking habits in 38-year-old
European men. Am. J. Epidemiol., 133, 257-265.

WASHBURN, R.A. & MONTOYE, H.J. (1986). The assessment of

physical activity by questionnaire. Am. J. Epidemiol., 123,
563-576.

WHITTEMORE, A.S., WU-WILLIAMS, A.H., LEE, M., SHU, Z., GAL-

LAGHER, R.P., DENG-AO, J., LUN, Z., XIANGHUI, W., KUN, C.,
JUNG, D., TEH, C.Z., CHENGDE, L., YAO, J.X., PAFFENBARGER,
R.S. Jr. & HENDERSON, B.E. (1990). Diet, physical activity, and
colorectal cancer among Chinese in North America and China. J.
Natl Cancer Inst., 82, 915-926.

				


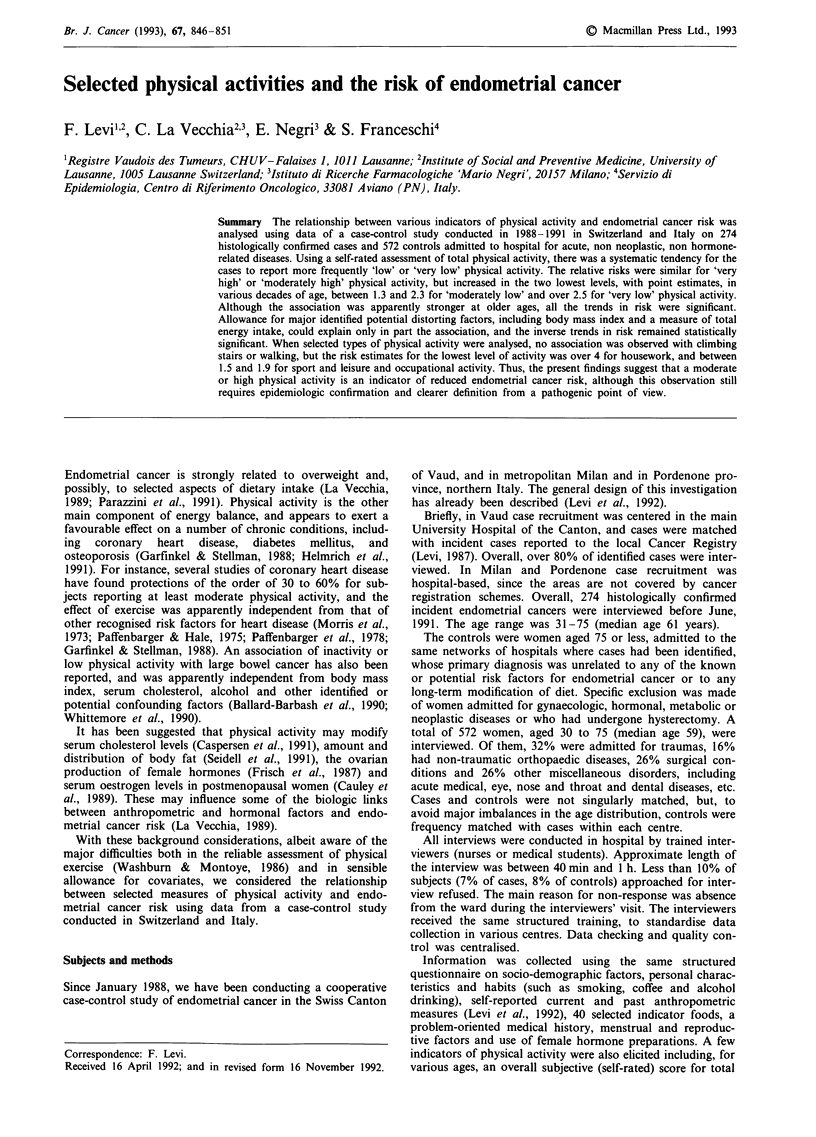

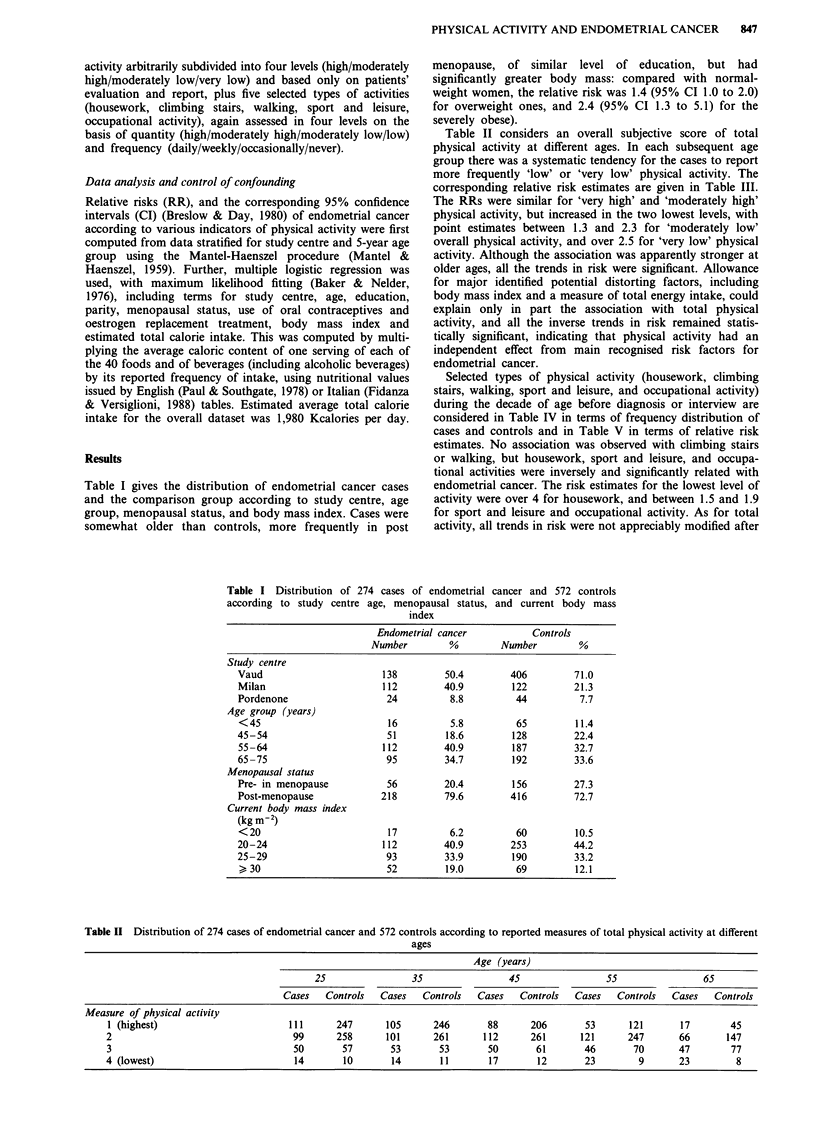

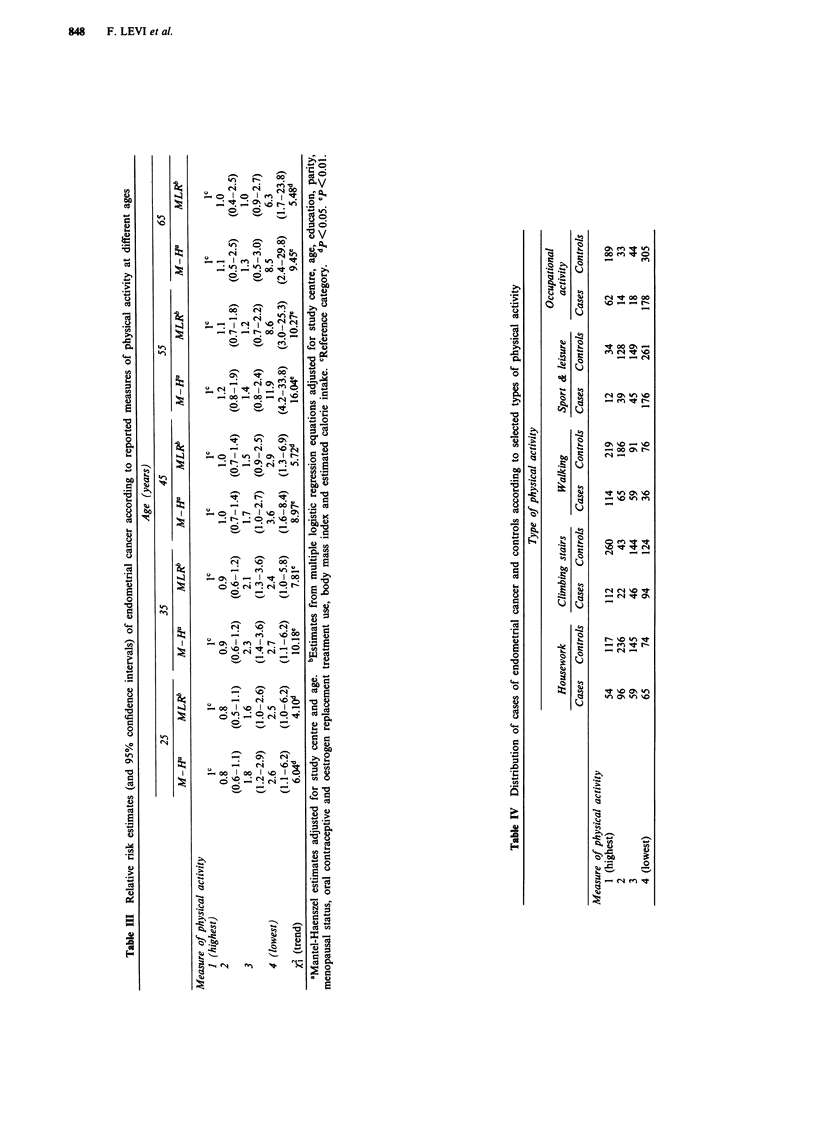

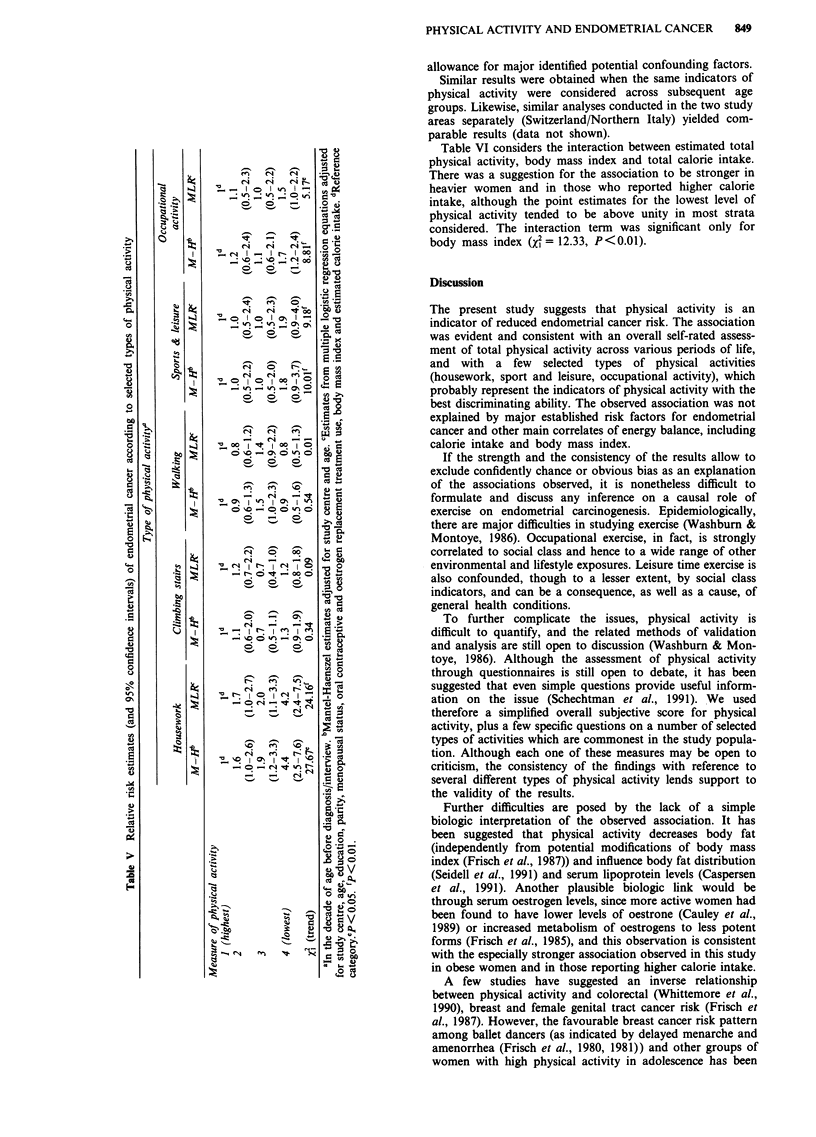

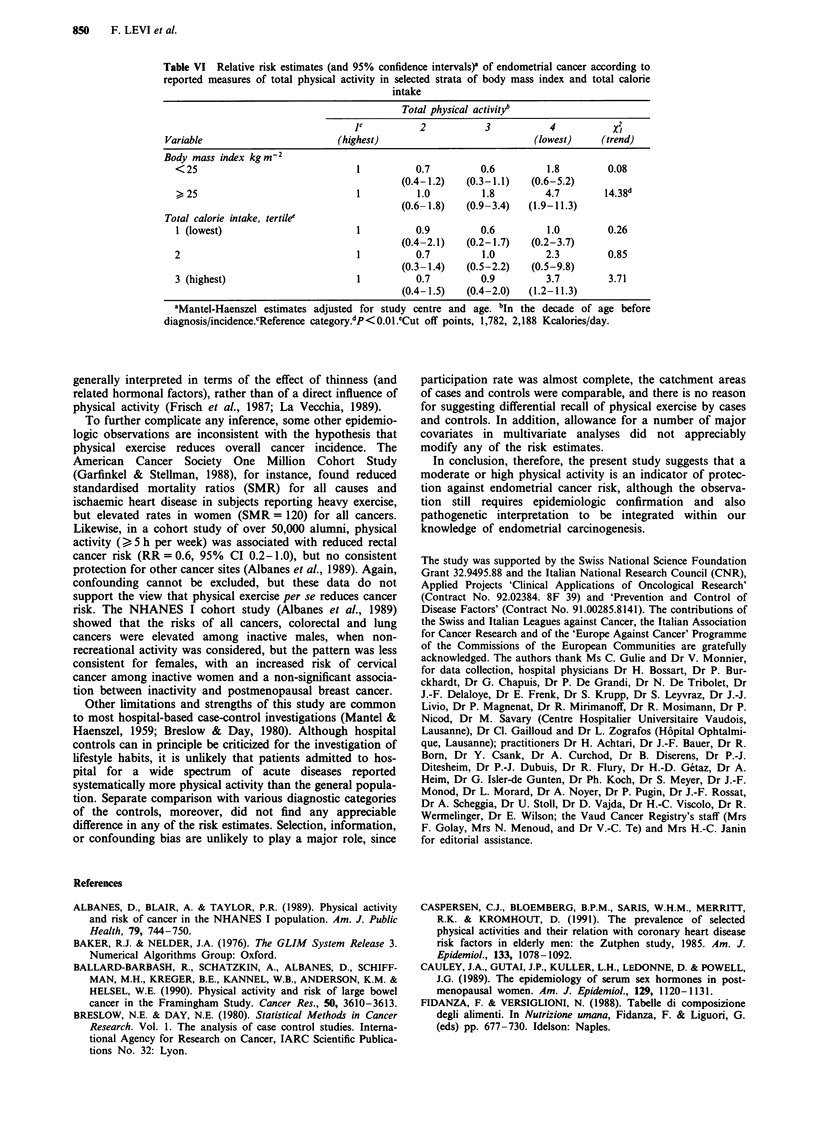

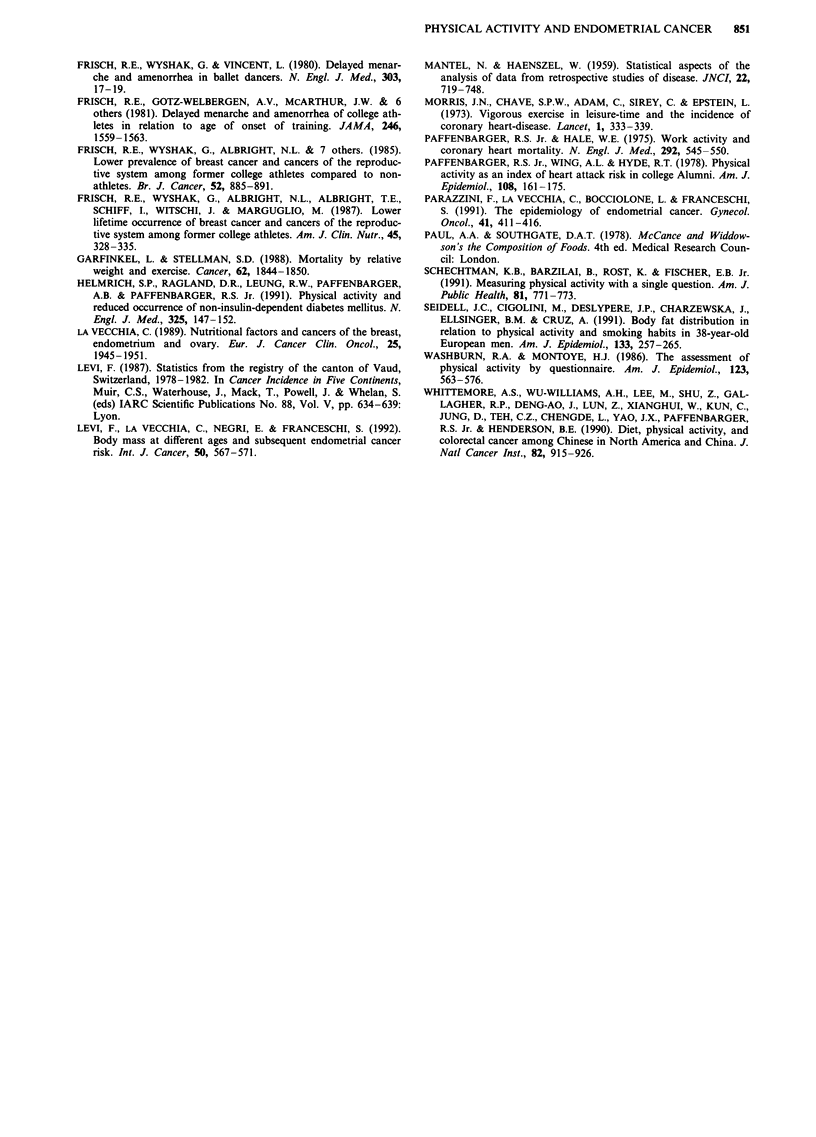

